# Similarities in semantic processing across verbal and pictorial domains in school children with developmental language disorder

**DOI:** 10.3389/fpsyg.2025.1548289

**Published:** 2025-04-01

**Authors:** Hanna Lindfors, Kristina Hansson, Neil Cohn, Annika Andersson

**Affiliations:** ^1^Department of Swedish, Linnaeus University, Växjö, Sweden; ^2^Department of Clinical Sciences, Lund University, Lund, Sweden; ^3^Department of Communication and Cognition, Tilburg University, Tilburg, Netherlands

**Keywords:** semantic processing, developmental language disorder (DLD), domain general, event-related potentials (ERPs), N400

## Abstract

This study investigates whether Developmental Language Disorder (DLD) is a specific language impairment or a domain-general disorder, thereby addressing the broader question of whether language processing is distinct from or comparable to cognitive processing in other domains. Specifically, we investigate semantic processing in verbal and pictorial domains among 9–12-year-old children with DLD in comparison to an age-matched control group. We measured the amplitude of the event-related potential (ERP) effect indicating semantic processing, the N400, to narratives in the form of both auditorily presented sentences and of wordless picture sequences (comic strips). We compared the N400 effect of predictability in both domains across group. Our findings from a total of 39 participants show an expected N400 effect in both domains in age-matched controls, though with longer latency for the more unfamiliar picture domain but no N400 effect in either domain in children with DLD. This study, thus, indicates similarities in semantic processing across the verbal and the pictorial domains in children with DLD, which is consistent with domain general theories of language.

## 1 Introduction

The issue of whether language processing is a specialized mechanism unique to language or a domain-general operation analogous to cognitive processes in other domains remains unresolved. One approach to address this issue is to study children with Developmental Language Disorder (DLD) (Leonard, 2014), which modular views of language claim is domain specific, and thus language would be the only affected area (e.g., Van der Lely, 2005). Conversely, if children with DLD exhibit broader difficulties, then domain-general views of language would be reinforced (e.g., Emergentism: Evans, 2001; Neuroconstructivism: Karmiloff-Smith, 2009). To address this theoretical issue in the present study, we compare semantic processing of non-verbal picture sequences (comic strips) conveying wordless narratives to semantic processing of utterances conveying verbal narratives in children with DLD in comparison with age-matched controls with typical language development.

The condition DLD involves long-lasting language learning difficulties and below average language comprehension and production, that impair communication in everyday life (Bishop et al., 2016; Bishop et al., 2017; World Health Organization, 2019). Criteria for DLD in research studies have required typical non-verbal IQ (i.e., no more than 2 SD below the population mean; Calder et al., 2022; Norbury et al., 2016). Even so, meta-analyses demonstrate that children with DLD have lower scores on behavioral measures of non-verbal cognition than children with typical language development. For instance, Gallinat and Spaulding (2014) found differences in non-verbal IQ, Pauls and Archibald (2016) in inhibitory control and cognitive flexibilty, and Vugs et al. (2013) in visuospatial working memory. Studies of visual narratives specifically have also implied that sequential image understanding is affected by DLD, as they score lower on non-verbal tasks like picture arrangement (Baker, 2013; Holmes, 2001; Watkins et al., 2002). The findings from these behavioral studies, suggesting that the verbal domain is not uniquely affected in DLD, could challenge domain-specific views of language.

However, behavioral studies of non-verbal cognition involve tasks that can be influenced by children’s language proficiency. Even if both behavioral task instructions and responses are non-verbal, children with DLD may not use inner speech (verbal thinking) as efficiently as children with typical language development when solving the tasks (Alderson-Day and Fernyhough, 2015; Maryniak, 2022; Nedergaard et al., 2023). Low scores on behavioral measures of non-verbal cognition in children with DLD may therefore be attributed to the behavioral tasks being biased by the language difficulties in children with DLD. Addressing the issue of domain specificity versus generality through behavioral studies of children with DLD thus presents considerable challenges.

The challenges with behavioral studies of non-verbal processing in children with DLD can be avoided by using neural measures, such as event-related potentials (ERPs) that do not require any behavioral response. Accordingly, neural measures could be thought of as less biased than behavioral measures. In the present study, we measure the ERP response termed the N400 (a negative-going voltage deflection around 400 ms after the onset of for instance words or pictures) as it is an established neural marker of semantic processing (Kutas and Federmeier, 2010; Kutas and Hillyard, 1980). The N400 has for a long time been known to be elicited across domains, including but not limited to stimuli such as words, pictures, and environmental sounds (e.g., Ganis et al., 1996; Van Petten and Rheinfelder, 1995). The amplitude of the N400 is larger (i.e., more negative) for less predictable stimuli than for more predictable stimuli and this amplitude difference is termed the N400 effect (Bornkessel-Schlesewsky and Schlesewsky, 2019; Coderre et al., 2020; Kutas and Federmeier, 2010).

In the two following sections, we review previous studies about the N400 effect in children with DLD and the N400 effect for narratives across populations. We then specify the present study and our research questions. The review will reveal that semantic processing of isolated sentences is affected in younger children with DLD. However, no studies have examined the neurocognition of their semantic processing of narratives, whether in the form of consecutive sentences or non-verbal picture sequences. This study targets this gap and thus offers a novel comparison of semantic processing across verbal and pictorial domains in children with DLD.

### 1.1 Semantic processing in children with DLD as indicated by the N400 effect

Several studies have examined semantic processing in children with DLD by measuring the N400 effect (Table 1). These studies indicate that the mean amplitude of the N400 effect differs between children with DLD below the age of 13 years and peers with typical language development when the stimuli consist of sentences. An example of this type of stimulus is *Barbie bakes the bread/people in the kitchen* (Fonteneau and van der Lely, 2008). The atypical mean amplitude of the N400 effect for sentences has consisted of either an absent N400 effect (i.e., the mean amplitude of the N400 was not larger for unpredictable stimuli than for predictable stimuli) or a greater mean amplitude of the N400 effect (Neville et al., 1993; Pijnacker et al., 2017; Popescu et al., 2009; Sabisch et al., 2006). Although the N400 effect was absent in a traditional time-window (300–500 ms) in the study by Pijnacker et al. (2017), the N400 effect was present in a later time-window (500–800 ms). An N400 effect with longer latency suggests slower semantic processing in this group. Unlike these studies with preschoolers and school children^1^ with DLD, studies with teenagers with DLD do not suggest differences in mean amplitude compared to teenagers with typical language development (Haebig et al., 2017; Weber-Fox et al., 2010). In studies in which teenagers and school children are lumped together into a single sample (Courteau et al., 2023a; Cummings and Čeponienė, 2010; Evans et al., 2022; Fonteneau and van der Lely, 2008; Kornilov et al., 2015), three out of the five studies show no difference between children with DLD and children with typical language development. These inconsistencies are problematic to interpret given the wide age range of the participants.

**Table 1:** Summary of reviewed studies, sorted by age group.

Study	Age group	N_DLD_, N_TD_	Stimuli	N400 effect
Pijnacker et al. (2017)	Preschoolers 5;2 (0;6) 4;2–6;5	37, 25	Spoken sentences	Only for TD 300–500 ms, but for both groups 500–800 ms
Haebig et al. (2018)	Preschoolers 4;11 (0;7)	15, 15	Picture-word pairs	For both groups and no group difference in mean amplitude
Popescu et al. (2009)	School children 7;0 (0;9) 6–8	8, 7	Spoken sentences	Only for TD pre language intervention^1^, but for DLD post language intervention
Neville et al. (1993)	School children 8–10	12^2^, 12	Written sentences	For both groups, but greater mean amplitude for DLD than TD
Sabisch et al. (2006)	School children 9;7 (1;9)	16, 16	Spoken sentences	Only for TD
Weber-Fox et al. (2010)	Teenagers 16;0 (0;3) 14;3–18;1	15, 15	Spoken sentences	For both groups and no group difference in mean amplitude
Haebig et al. (2017)	Teenagers 16;5 (1;5)	19, 18^3^	Spoken sentences	For both groups and no group difference in mean amplitude
Kornilov et al. (2015)	Mixed 10;5 (2;3) 7;2–15;8	23, 16	Picture-word pairs	For both groups and no group difference in mean amplitude 310–410 ms, but smaller for DLD than TD 410–600 ms
Cummings and Čeponienė (2010)	Mixed 11;5 (2;6) 7–15	16, 16	Picture-word pairs Picture-sound pairs (i.e., non-verbal)	For both groups and no group difference in mean amplitude
Courteau et al. (2023a)	Mixed 14;1 (0;7) 12–15	17, 19	Spoken sentences paired with single pictures	For both groups and no group difference in mean amplitude, but an earlier onset for TD than DLD
Fonteneau and van der Lely (2008)	Mixed 14;3 10–21	18, 18^4^	Spoken sentences	For both groups and no group difference in mean amplitude
Evans et al. (2022)	Mixed 15;2 (2;2) 11;11–18;11	14, 14	Spoken sentences	For both groups and no group difference in mean amplitude

The term SLI has been used in previous research. We use the broader term DLD, for consistency with current terminology (Bishop et al., 2016; World Health Organization, 2019). Age in years; months. Mean, standard deviation within brackets, and age range below when available. TD: Control group of age-matched children with typical language development. ^1^The language intervention was designed to improve “ability to comprehend, retell, and generate multiepisode stories and to use complex grammar.” ^2^A larger group of children (*n* = 22) was included in a part of the study that did not examine semantic processing. ^3^A third group, referred to as “SLI-recovered,” were also part of the study (*n* = 15). ^4^A group of younger children (*n* = 20) was also included in the study as a language-matched control group.

In studies of both teenagers and younger children, the most frequent type of stimuli is auditorily presented sentences. A few studies have used single pictures paired with single words (Cummings and Čeponienė, 2010; Haebig et al., 2017; Kornilov et al., 2015). To the best of our knowledge, there is only one ERP study with children with DLD that examined semantic processing using non-verbal stimuli (Cummings and Čeponienė, 2010). Cummings and Čeponienė (2010) presented participants with both non-verbal stimuli (picture-sound pairs) and verbal stimuli (picture-word pairs). The pictures were colored photos of real objects (e.g., a rooster). Each picture was, after a 600 ms delay, accompanied by a matching or mismatching auditorily presented word (e.g., *crowing* or *chiming*) or a matching or mismatching environmental sound (e.g., *cock-a-doodle-do* or *ding-dong*). The participants’ task was to press a button to indicate if the word or the sound matched or mismatched the picture. Mean amplitudes of N400 effects for the verbal stimuli and the non-verbal stimuli were measured from difference waves (matching minus mismatching) over 15 medial electrodes (placed over frontal, central, and parietal sites) in the typical N400 time-window (300–500 ms) and in four shorter time intervals (300–400, 400–500, 500–600, and 600–700 ms). Participants with DLD (labeled developmental language impairment by Cummings and Čeponienė, 2010) were 7- to 15-year-olds and had a non-verbal IQ score of 80 or higher, an expressive language score of 1.5 or more standard deviations below the mean on CELF-3 (Semel et al., 1995), and no other neurodevelopmental disorder with the exception for one participant who had Attention Deficit Hyperactivity Disorder (ADHD). The results showed no overall significant effect of stimulus type (verbal/non-verbal) or group (DLD/age-matched controls) and no interactions, suggesting similar mean amplitudes of N400 effects in both groups for verbal and non-verbal stimuli (Cummings and Čeponienė, 2010). Accordingly, the only study of semantic processing of non-verbal stimuli in children with DLD did not show differences compared to their peers. However, the age range was large (7–15 years). It is therefore unclear if children with DLD below the age of 13 years have typical or atypical semantic processing of non-verbal stimuli. No studies that we know of have used pictures exclusively. Semantic processing in the pictorial domain has thus not yet been investigated in children with DLD.

### 1.2 Semantic processing of narratives across populations

As shown in the previous section, studies have not examined semantic processing of narratives in children with DLD, either verbal narratives or visual narratives. Semantic processing of narratives, as indicated by the N400 effect, has however been investigated in other populations. Findings have suggested that there is no fundamental difference in adults’ semantic processing of a word in a sentence context and a verbal narrative context (van Berkum et al., 1999). However, narrative contexts can facilitate semantic processing. For example, sentences with animacy violations as “the peanut was in love” were more easily processed by adults than sentences as “the peanut was salted” when presented in narratives (Nieuwland and Van Berkum, 2006). There are studies of semantic processing of verbal narratives in children (Levari and Snedeker, 2024; Lindfors et al., 2024; Weber-Fox et al., 2013), but studies of adults are dominating.

Interestingly, visual narratives have elicited the same ERP effects as in sentence processing, both to the manipulations of the semantics and “grammar” of visual narratives (Cohn, 2020a). As with studies of verbal narratives, semantic processing of visual narratives has mainly been studied in adults (e.g., Coderre et al., 2020; Cohn et al., 2012; West and Holcomb, 2002). Not only neurotypical adults have been studied. Indeed, the N400 effect has been shown to be affected for both verbal and visual narratives in adults with autism (Coderre et al., 2018; Kubinski et al., 2024). Although a couple of studies have investigated semantic processing of visual narratives in children (Lindfors et al., 2024; Manfredi et al., 2020), it remains unknown whether the N400 effect for visual narratives is affected in children with DLD. Given claims that visual narratives are similar in their structure and processing to verbal narratives, investigation of semantic processing in children with DLD can provide insights into the supposed domain-specificity of language.

### 1.3 Present study

The present study addresses the gap in ERP research on semantic processing of non-verbal stimuli in children with DLD below 13 years, an age-group that has demonstrated atypical semantic processing of sentences in previous studies. Furthermore, semantic processing in the verbal domain and the pictorial domain are examined using a within-subjects design. These novel elements have the potential to contribute to an advanced understanding of whether the nature of language is specific or domain general. Indeed, by investigating whether DLD is a disorder specific to language or rather a domain-general disorder, we address the broader question of whether language processing is distinct from or comparable to cognitive processing in other domains. In both the verbal and the pictorial domain, we manipulate semantic predictability in narrative contexts. Narratives have higher ecological validity than isolated sentences, as everyday language processing does not involve isolated sentences without any context (Hasson et al., 2018). Verbal narratives allow for a greater build-up of semantic context in contrast to an isolated sentence and have been utilized in studies of semantic processing in children and adults, as reviewed above, but not with children with DLD. In the verbal domain, we present narratives in the form of auditorily presented consecutive sentences. In the pictorial domain, picture sequences that convey wordless narratives are presented panel by panel. This ERP paradigm, which we have previously used with children (Lindfors et al., 2024) and that has been used in several studies with adults (e.g., Coderre et al., 2018; Cohn et al., 2012), is especially interesting to use in the present study, as behavioral studies of children with DLD have suggested lower performance on non-verbal picture arrangement tasks (Baker, 2013; Holmes, 2001; Watkins et al., 2002). Picture arrangement tasks, where pictures must be ordered to make a meaningful sequence, are included in cognitive assessment batteries (WISC-III: Wechsler, 1991; KABC-II NU: Kaufman and Kaufman, 2018) and have been used to indicate for example sequential reasoning and social understanding but have also been referred to as a visual narrative task by Cohn (2020b). The lower performance on picture arrangement tasks could, however, be biased by the language difficulties in children with DLD, even though these tasks do not require overt verbal responses, as argued above. By using the ERP paradigm, we can measure processing of picture sequences in children with DLD without requiring any overt behavior, making the measure less biased.

We ask the following research questions:

*RQ1*. Does semantic processing differ between children with DLD and peers with typical language development?

*RQ2*. If so, is the difference in semantic processing between the groups affected by domain (verbal/pictorial)?

We hypothesize that semantic processing of consecutive sentences and picture sequences in 9-to 13-year-old children with DLD will differ from that in peers with typical language development. This hypothesis is based on the ERP studies of semantic processing of isolated sentences in children under 13 years with DLD and on the behavioral studies of their non-verbal cognition. Crucially, we hypothesize that semantic processing is domain general (i.e., similar in the verbal and pictorial domains) in children with DLD. This would replicate findings of a recent study (Lindfors et al., 2024) that included children with typical language development (who constitute part of the age-matched control group in the present study). We thus expect a main effect of group (typical language development/DLD) *without* an interaction with domain (verbal/pictorial).

More specifically, we expect the mean amplitude of the N400 effect (i.e., the mean amplitude of the N400 for predictable stimuli subtracted from that for unpredictable stimuli) to differ between the groups (typical language development/DLD) in one of the following ways. Children with DLD will either have an absent N400 effect in line with most studies (Pijnacker et al., 2017; Popescu et al., 2009; Sabisch et al., 2006) or a greater mean amplitude of the N400 effect as suggested by one study (Neville et al., 1993). Another possibility is that children with DLD do have an N400 effect like peers with typical language development, although with a longer latency (Pijnacker et al., 2017). If the N400 effect is delayed in children with DLD, we would see a main effect of group (typical language development/DLD) in the first time-window (300–500 ms) but not in the second time-window (500–700 ms).

## 2 Method

### 2.1 Participants

Sample size was determined based on sample sizes of previous studies (Table 1). Participants with and without DLD were recruited in Sweden through schools, web postings, and personal contacts. Of the 43 recruited children (DLD: 20, typical language development: 23) 4 were excluded from analyses: 1 with typical language development (TD) due to a technical error that hindered ERP data collection, and 3 participants with DLD since they opted to terminate their participation shortly after the sessions started. Participant characteristics of the final sample (TD: 22, DLD: 17) are presented in Table 2. All participants were reported having Swedish as a first language, normal hearing and vision, and no neurodevelopmental disorder other than DLD, except for two children with DLD who also had Autism Spectrum Disorder (ASD). In the presence of ASD, the term ‘Language Disorder Associated with ASD’ has been recommended instead of DLD (Bishop et al., 2017), but we use DLD in accordance with ICD (“An additional diagnosis of Developmental Language Disorder should not be assigned to individuals with Autism Spectrum Disorder based solely on pragmatic language impairment. However, both diagnoses may be assigned if there are additional specific impairments in semantic, syntactic and phonological development,” World Health Organization, 2019). Note that we previously have published data for a subset of the participants (Lindfors et al., 2024) that are used as age-matched controls in the present study.

**Table 2:** Participant characteristics.

	TD	DLD	*df*	*t*	*p*
Age	11;4 (0;11) 9;4–13;1	11;4 (0;8) 9;10–12;7	37	0.59	0.56
SES	5.7 (0.8) 4.0–7.0	4.8 (2.0) 1.0–6.5	27	1.67	0.11
Non-verbal IQ	55 (24) 5–98	34 (26) 8–88	36	2.56	0.02
Sentence recall	54 (25) 5–91	0.5 (1.2) 0.1–5	37	8.84	<0.001
VLFI total score	10.3 (6.2) 4.5–29	9.6 (6.3) 3–22	37	0.37	0.72
VLFI subquestions:					
Books	2.0 (1.5) 1–7	2.1 (1.1) 1–4	37	−0.14	0.89
Strips	2.0 (1.5) 1–7	1.7 (0.8) 1–3	37	0.60	0.55
Graphic novels	2.7 (1.6) 1–7	2.2 (1.5) 1–5	37	0.98	0.34
Manga (Japanese comics)	1.5 (1.2) 1–6	1.8 (1.5) 1–7	37	−0.65	0.52

Mean (standard deviation), range. Year; months. Socioeconomic status (SES): average parental education on a 7-point scale (Hollingshead, 1975), where 1 = less than 7 years of education and 7 = PhD or master. Non-verbal IQ: percentile scores on Raven’s 2 (Raven et al., 2018). Sentence recall: percentile scores on the subtest Recalling Sentences of the Swedish version of CELF-4 (Semel et al., 2013). VLFI: total score and scores on four questions of a Swedish translation and adaption of the Visual Language Fluency Index (VLFI) questionnaire (Cohn, 2014) on a 7-point Likert scale, where 1 = never and 7 = always.

**Figure 1:**
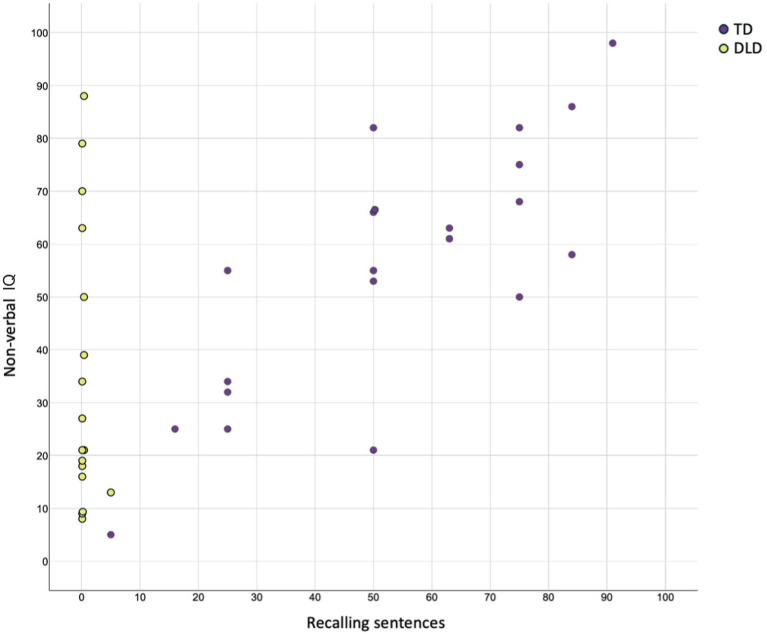
Scatterplot of participants’ percentile scores on standardized verbal and non-verbal behavioral measures. *X*-axis: sentence repetition measured with the subtest Recalling Sentences of the Swedish version of CELF-4. *Y*-axis: non-verbal IQ measured with Raven’s 2. The scatterplot shows individual differences and suggests that the verbal and non-verbal behavioral measures are positively correlated in the participants with TD, but unrelated in the participants with DLD.

To characterize the sample and ensure comparability between the DLD group and the age-matched control group, we provide measures of socioeconomic status (SES; parental educational level), experience of reading comics, and non-verbal IQ (Table 2), as these factors, like age and gender, may be associated with language proficiency or comics reading skill. The measure of SES was obtained by averaging parents’ rating of their educational level on a 7-point scale (Hollingshead, 1975). Experience of reading comics was measured with the Visual Language Fluency Index questionnaire (Cohn, 2014), that was adapted to children and translated into Swedish (see the Supplementary material in Lindfors et al., 2024). The questions were answered by participants through ratings on a 7-point Likert scale. The measure of non-verbal IQ was obtained with the digital short version of Raven’s Progressive Matrices (Raven’s 2, Raven et al., 2018), which according to the manual places demands on for example attention to visual detail, reasoning, fluid intelligence, and working memory. A computer error hindered the administration of Raven’s 2 for one of the participants with TD. This participant was included in the analyses as ERP data had been collected successfully. As shown in Table 2, the groups (TD, DLD) were matched on group means of age, socioeconomic status, and comics reading frequency. There was an expected statistically significant difference between groups on the measure of non-verbal IQ though all individuals in both groups scored above the threshold for Disorder of Intellectual Development (i.e., two *SD* below the population mean). However, ranges (Table 2 and Figure 1) suggest large individual differences on the measure of non-verbal IQ for both groups.

To confirm the grouping of participants with TD and DLD, we administered a verbal task. The verbal task was the Recalling Sentences subtest from the Swedish version of the Clinical Evaluation of Language Fundamentals (CELF-4, Semel et al., 2013), in which participants are required to repeat sentences with increasing length and complexity. A sentence repetition task was selected, as sentence repetition has been shown to be a clinical marker of DLD in several languages (Conti-Ramsden et al., 2001; Courteau et al., 2023b; Taha et al., 2021; Thordardottir and Brandeker, 2013). As expected for CELF-4 Recalling Sentences, all participants with DLD scored below the average for their age according to the Swedish norms (Table 2, Figure 1). More specifically, all participants with DLD scored lower than 1.5 standard deviations below the mean for their age group. The participants with DLD thus scored below frequently used thresholds of at least 1 or 1.5 standard deviations below the population mean on verbal measures (Bishop, 2014; who points out that it is an arbitrary criterion).

### 2.2 Stimuli

**Figure 2:**
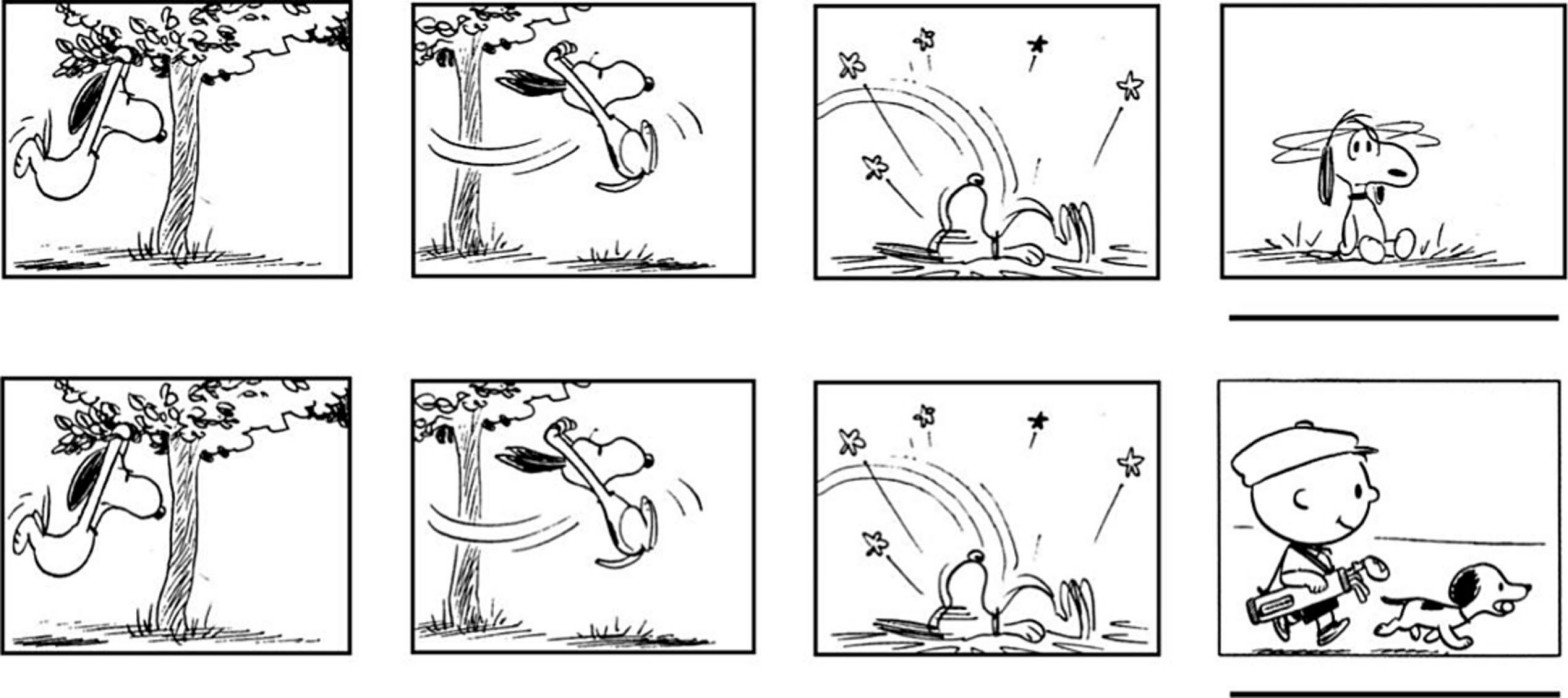
Illustration of two versions of a part of a picture sequence. The stimuli were developed by Cohn et al. (2014). Reproduced with permission. Pictures are copyright Peanuts Worldwide. The underlining indicates the critical picture for each condition (predictable/unpredictable). A participant never saw both versions of a picture sequence.

Stimuli consisted of auditorily presented sentences that conveyed verbal narratives and picture sequences (wordless comic strips presented panel by panel) that conveyed non-verbal narratives (see Lindfors et al., 2024, for a detailed description of the stimuli). Stimuli were presented to participants with PsychoPy (Peirce et al., 2019). Examples from the verbal narratives are “The daddy penguin continues to bake cake in the kitchen” and “The daddy penguin continues to bake nose in the kitchen,” which are translations from the original versions in Swedish (“Pappapingvinen fortsätter baka kaka i köket,” “Pappapingvinen fortsätter baka näsa i köket”). The underlining indicates the critical words for each condition (predictable/unpredictable). The predictable critical words are congruent with the complete semantic context (i.e., verbal narratives accompanying animations of a penguin family). A participant never heard both versions of a sentence. An illustration of examples from the pictorial narratives is provided in Figure 2. The original stimuli were developed by Cohn et al. (2014) out of *Peanuts* comic strips made by Charles M. Schulz. To isolate the impact of the context on ERP responses and avoid effects related to specific words and pictures, we used the same words across both conditions (predictable/unpredictable) for the verbal domain and the same pictures across both conditions for the pictorial domain.

### 2.3 General procedure

The procedure followed that in the Lindfors et al. (2024) study with the exception that some of the children with TD and all the children with DLD took part in the study at their school instead of at the Linnaeus University. Like for the Lindfors et al. (2024) study, participants and their caregivers provided written informed consent that was approved by the Swedish Ethical Review Authority.

### 2.4 EEG recordings, processing, and analyses

The EEG recordings and processing were equivalent to that in the Lindfors et al. (2024) study, including the use of BrainProducts ActiCap and EEGLAB (Delorme and Makeig, 2004) in MATLAB (The MathWorks Inc, 2020). Time-windows and electrodes used in analyses were selected based on previous research and prior to inspection of grand average waveforms and topographic plots, to avoid circular analysis (Makin and Orban de Xirvy, 2019) and “bogus effects” (Luck and Gaspelin, 2017). The 300–500 ms time-window was selected in accordance with previous N400 studies with adults (reviewed by Šoškić et al., 2022) and children (summarized in Table 1). As previous studies with children included an additional later time-window or an extended time-window (Haebig et al., 2018; Neville et al., 1993; Pijnacker et al., 2017; Sabisch et al., 2006), we also included a later time-window (500–700 ms). The electrodes (left, medial, and right over frontal sites F3/Fz/F4; over central sites C3/Cz/C4; over posterior sites P3/Pz/P4) were selected based on the review study by Šoškić et al. (2022), which showed that these electrodes are the most frequently used electrodes in N400 studies.

To answer our research questions, we measured the mean amplitude for predictable and unpredictable words and pictures for each of the two time-windows (300–500 ms, 500–700 ms). These measures were subjected to repeated measures ANOVA with the following four within-subject factors: *domain* (verbal/pictorial), *predictability* (predictable/unpredictable), *lateral* (left/medial/right), and anterior–posterior position aka *Ant/Post* (frontal/central/posterior). The between-subjects factor was *group* (TD/DLD). Interactions between domain and/or predictability and electrode position factors in omnibus ANOVAs motivated additional analyses on data within group and domain. Those step-down analyses used Bonferroni-corrected *p-*values based on the number of analyses that were considered separately. The Greenhouse–Geisser correction was applied to all statistics with more than two levels of a factor. Corrected *p*-values and uncorrected degrees of freedom are reported. Effect sizes for the ERP effects are reported as partial eta squared (*η^2^*) values.

We expected a difference in semantic processing between children with DLD and peers with TD (RQ1), which would be demonstrated by a main effect of group. We also anticipated similar processing across domains within each group and, thus, that semantic processing would differ between the groups in both the verbal and the pictorial domain (RQ2). This would be suggested by a difference in semantic processing between the groups that is unaffected by domain (i.e., no interaction between group and domain).

## 3 Results

**Figure 3:**
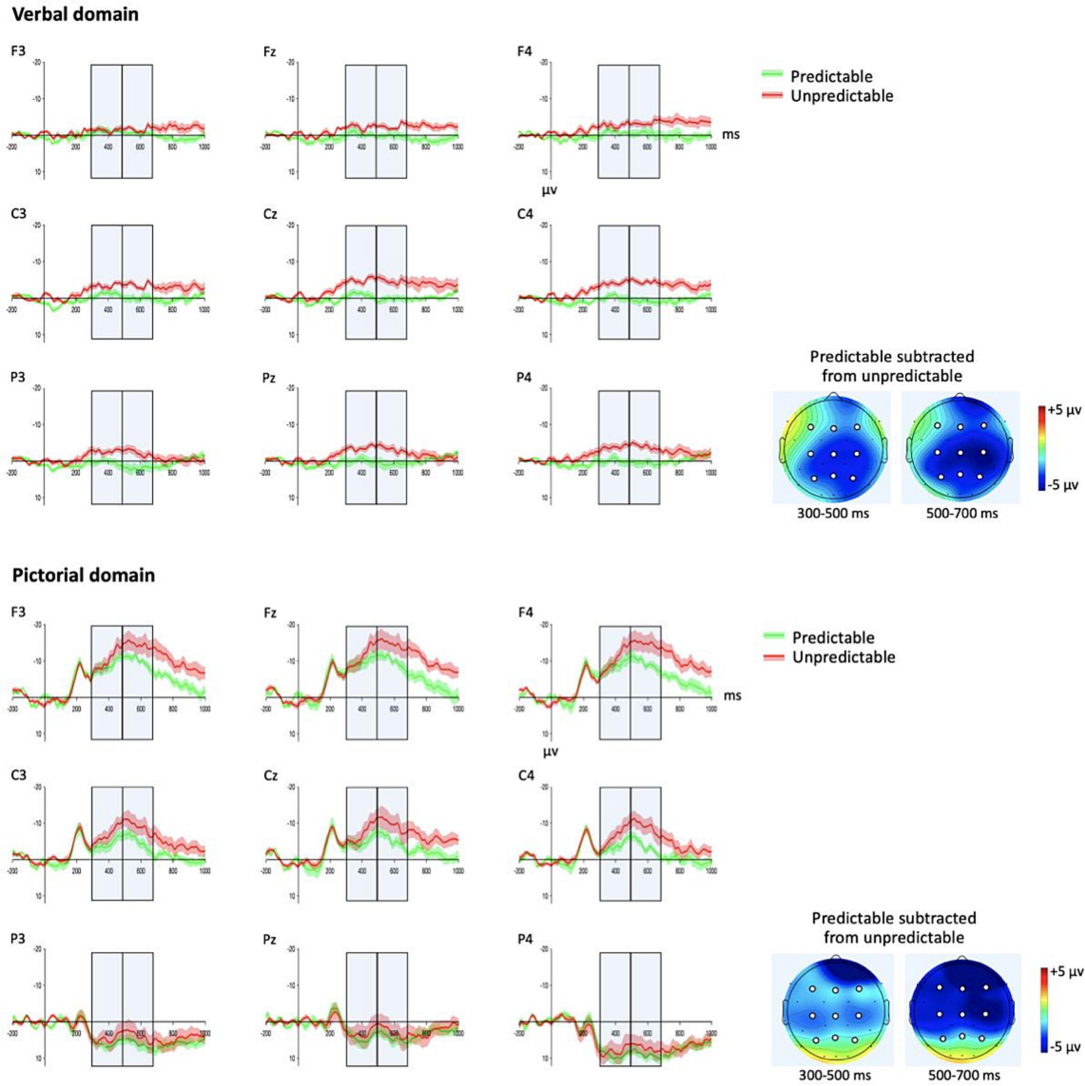
TD participants’ grand average waveforms and topographic plots for the verbal and the pictorial domain. Gray boxes: time-windows (300–500 ms, 500–700 ms) used in the statistical analyses. Green waveforms: predictable stimuli in its narrative context. Red waveforms: unpredictable stimuli in its narrative context. Shadings of the waveforms: standard error. Topographic plots: mean amplitude difference (predictable subtracted from unpredictable) in the two time-windows. White circles: electrodes (F3, Fz, F4, C3, Cz, C4, P3, Pz, P4) used in the statistical analyses (Table 3).

**Figure 4:**
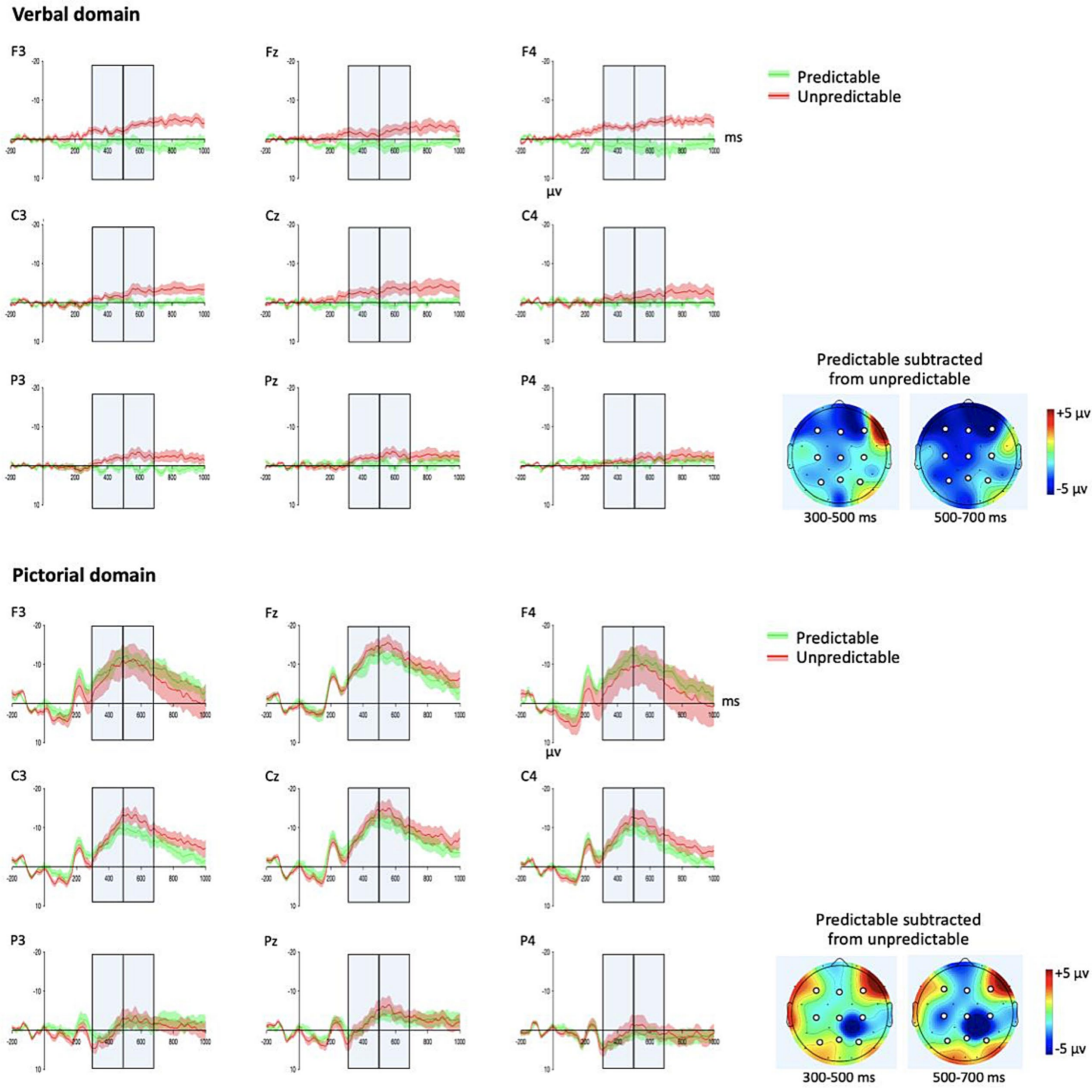
Developmental language disorder (DLD) participants’ grand average waveforms and topographic plots for the verbal and the pictorial domain. Gray boxes: time-windows (300–500 ms, 500–700 ms) used in the statistical analyses. Green waveforms: predictable stimuli in its narrative context. Red waveforms: unpredictable stimuli in its narrative context. Shadings of the waveforms: standard error. Topographic plots: mean amplitude difference (predictable subtracted from unpredictable) in the two time-windows. White circles: electrodes (F3, Fz, F4, C3, Cz, C4, P3, Pz, P4) used in the statistical analyses (Table 3).

In both time-windows, and especially for children with DLD, there was a stronger negativity for the pictorial domain compared to the verbal domain (Figures 3, 4 and Table 3) presumably indicating that children in both groups were less familiar with the pictorial domain than the verbal domain. The distribution of the effects, also in both time-windows and disregarding predictability, suggested more fronto-central and left-medial distribution for the pictorial domain while more wide-spread effects for the verbal domain. While there was a strong effect of predictability across groups and domains in both time-windows (Table 3) the interaction with domain and predictability in the first time-window suggested a shorter latency for the N400 effect of predictability for the verbal domain.

**Table 3:** Repeated measures ANOVA.

	300–500 ms				500–700 ms		
	*df*	*F*	*p*	*η^2^*	*F*	*p*	*η^2^*
Domain	1,37	11.925	0.001	0.24	28.011	<0.001	0.43
Domain × Group	1,37	4.329	0.044	0.11			
Domain × Predictability	1,37	5.307	0.027	0.13			
Domain × Predictability × Group	1,37	2.546	0.032	0.13			
Domain × Lateral	2,74	16.054	<0.001	0.30	19.680	<0.001	0.35
Domain × AntPost	2,74	82.399	<0.001	0.69	82.123	<0.001	0.69
Domain × AntPost × Group	2,74	3.216	0.074	0.08	4.543	0.031	0.11
Predictability	1,37	17.106	<0.001	0.32	30.452	<0.001	0.45

Domain (verbal/pictorial), Predictability (predictable/unpredictable), Lateral (left/medial/right), AntPost (frontal/central/posterior), and group (TD/DLD). Nine electrodes (F3, Fz, F4, C3, Cz, C4, P3, Pz, P4) were used in the statistical analyses. Only statistics that include Domain or Predictability are included.

Table 3 demonstrates two significant interactions that included domain and group (300–500 ms, domain × predictability × group; 500–700 ms, domain × ant/post × group). We followed up these interactions for each group within each domain (Table 4) with the corrected alpha level at 0.0125. These analyses showed an N400 effect (i.e., stronger negativity for unpredictable than predictable) in children with TD in both time-windows in the verbal domain and with a later onset (500–700 ms) and strongest over frontal electrode sites in the pictorial domain (Figure 3). In contrast, the N400 effect of predictability for the verbal domain in children with DLD did not reach significance in either time-window but was approaching significance (both *p*s = *0*.053) when not considering corrected alpha levels (Table 4 and Figure 4). There were no effects in children with DLD for the pictorial domain (300–500 ms, all *p*s > 0.609; 500–700 ms, all *p*s > 0.242).

**Table 4:** Follow up analyses by group and domain.

Domain	Group			300–500 ms			500–700 ms		
			*df*	*F*	*p*	*η^2^*	*F*	*p*	*η^2^*
Verbal									
	TD								
		Predictability	1,21	28.842	<0.001^*^	0.58	36.198	<0.001^*^	0.63
	DLD								
		Predictability	1,16	4.371	0.053	0.22	4.371	0.053	0.22
Pictorial									
	TD								
		Predictability	1,21	3.757	0.066	0.15	11.258	0.003^*^	0.35
		Predictability × AntPost	2,42				7.480	0.008^*^	0.26

*Significant with corrected alpha level, 0.0125. Only statistics including Predictability are included.

## 4 Discussion

Addressing the issue of whether language processing is domain specific or domain general, this study provides novel insights through a unique investigation of semantic processing across the verbal and the pictorial domain in 9– to 12-year-old children with DLD in comparison to an age-matched control group. Measures of neural activity for consecutive sentences and wordless picture sequences suggest that atypical semantic processing may not be specific for the verbal domain in school children with DLD. The school children with DLD did not have a statistically significant N400 effect in either the verbal domain or the pictorial domain. In contrast, the age-matched control group had a statistically significant N400 effect in both domains. That semantic processing is affected across domains in DLD aligns with and extends meta-analyses of behavioral studies that have suggested differences between children with DLD and peers with typical language development beyond the verbal domain (Gallinat and Spaulding, 2014; Pauls and Archibald, 2016; Vugs et al., 2013). Accumulating evidence are thus consistent with domain general perspectives of language processing. In the following, we discuss our result in relation to previous ERP studies and offer potential implications.

**Figure 5:**
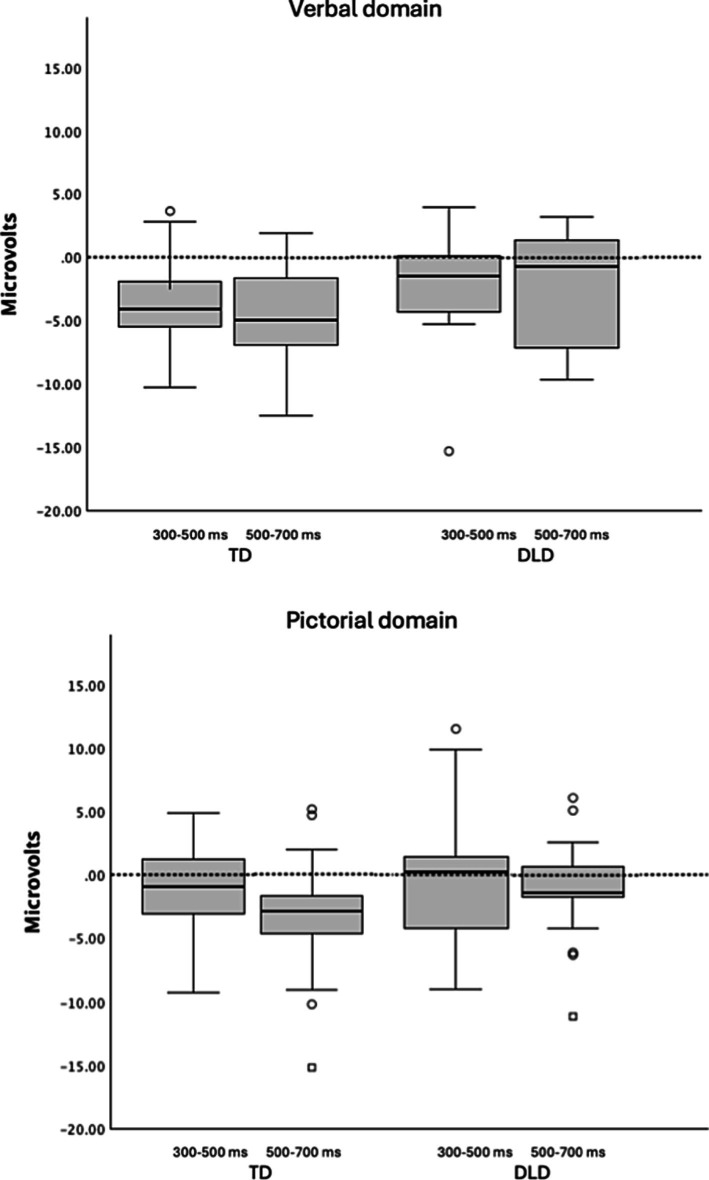
Boxplots of mean amplitude differences per group and domain over the nine electrodes that were used in the analyses. Time windows are given underneath. TD refers to children with typical language development. DLD refers to children with developmental language disorders. Boxes represent data within the interquartile range (IQR). Lines inside the boxes indicate the median. Whiskers represent data within 1.5 x IQR. Data points outside the whiskers are considered outliers.

The difference between the groups consisted of an expected N400 effect in both the verbal and the pictorial domains in the age-matched control group but no N400 effect in either domain in children with DLD, as evident in Figures 3–5. In children with DLD, the effect was approaching significance in the verbal domain (if disregarding corrections of alpha levels for multiple analyses) but not in the pictorial domain. This is the opposite of what would have been expected if DLD was specific to language. Had DLD been specific to language, we would have found a similar N400 effect in children with DLD as in the control group for the pictorial domain. The opposite tendency, however, only approached significance without corrections of alpha levels for multiple analyses. We therefore refrain from speculating on interpretations of this non-significant result.

The absence of N400 effects in children with DLD were in line with several previous studies demonstrating an absent N400 effect in the verbal domain in preschoolers and school children with DLD (Pijnacker et al., 2017; Popescu et al., 2009; Sabisch et al., 2006). However, these findings diverge from two studies of preschoolers and school children with DLD (Haebig et al., 2018; Neville et al., 1993). These two studies reported the presence of an N400 effect. A possible explanation for the contrasting findings is the employment of different stimuli. All the studies that did demonstrate an absent N400 effect in preschoolers and school children with DLD (Pijnacker et al., 2017; Popescu et al., 2009; Sabisch et al., 2006) utilized stimuli in the form of spoken sentences. In contrast, the study by Haebig et al. (2018) utilized single words paired with single pictures, which arguably are less complex than sentences. The less complex stimuli may be easier to process for children with DLD, which can explain the presence of an N400 effect in the study by Haebig et al. (2018). The presence of an N400 effect in the study by Neville et al. (1993) can however not be explained by a reduced complexity of the stimuli because written sentences were used. The finding of the Neville et al. (1993) study is unusual as all the other studies that used sentences demonstrated an absent N400 effect in preschoolers and school children with DLD. One explanation could be that the school children in the Neville et al. (1993) study may have had milder language impairments. However, this is speculative and the differences are difficult to explain. The present results extend previous findings from isolated sentences to consecutive sentence accompanying animations. This richer context could be considered as more ecologically valid. The most crucial extension is the comparison between the verbal and the pictorial domains. Although a few studies have presented single pictures combined with single words or sounds to children with DLD (Cummings and Čeponienė, 2010; Haebig et al., 2018; Kornilov et al., 2015), no study compared their semantic processing between the verbal and the pictorial domains. In summary, the present study not only corroborates the absent N400 effect in school children with DLD for complex verbal stimuli, but also extends this finding to the pictorial domain. These results suggest similarities in semantic processing between the domains in children with DLD. Thus, not only children with typical language development demonstrate similarities in semantic processing between domains (Lindfors et al., 2024).

Furthermore, it could be considered a limitation of this study that the wordless picture sequences may have been verbalized internally by participants. If so, their inner speech may have affected the semantic processing in the pictorial domain. Although this cannot be ruled out with the present design, we consider it unlikely that this has affected the results. The key idea in ERP research is that time-locked activity to specific events is enhanced through averaging across trials and participants, while non-time-locked activity is averaged out. Our ERP measures were time-locked to the onset of pictures. Since internal speech would differ in timing across trials and participants, its contribution would be averaged out and the waveform of the N400 would then not show the peaks that currently can be seen in Figures 3, 4. Inner speech is thus unlikely to systematically contribute to the ERP measures, leaving the effects driven by the pictures.

Implications of the similarities in semantic processing between the verbal and pictorial domains involve both theoretical and practical considerations for DLD. Theories that position DLD as a manifestation of general processing difficulties (e.g., Evans, 2001; Karmiloff-Smith, 2009) may have greater explanatory power than linguistic accounts (e.g., Van der Lely, 2005). Considerations of general processing difficulties may be warranted in clinical practice and should be investigated in future studies. Another important topic for future studies is to investigate if pictures serve the often-assumed compensatory function for children with DLD. It has been suggested that pictures are not easier to understand than language, especially in clinical populations (Coderre, 2020). If semantic processing is equally challenging in pictorial and verbal domains for children with DLD, then the provision of pictures is not inherently facilitative. Adjustments that typically are provided alongside pictures are potentially more crucial. For example, a reduction of the representational complexity, from lengthy sequences to segmented units, could be a critical factor for accessible communication. By focusing on different degrees of representational complexity, future research may offer a more comprehensive understanding of how children with DLD process representations across different domains. Ultimately, this could contribute to enhanced clinical and educational practices for children with DLD.

## 5 Conclusion

There was not a statistically significant N400 effect of semantic predictability in school children with DLD in either the verbal or the pictorial domain. In contrast, there were N400 effects in both domains in peers with typical language development. Crucially, the study reveals that semantic processing can be equally challenging in verbal and pictorial domains for school children with DLD. This finding is consistent with domain-general perspectives of language, rather than domain-specific perspectives that would predict a uniquely affected language module. The similarities in semantic processing between the verbal and pictorial domains in school children with DLD challenge the assumption that pictures are inherently easier to understand than language.

## Data Availability

The raw data supporting the conclusions of this article will be made available by the authors, without undue reservation.

## Footnotes

^1^By *school children* we are referring to children below the age of 13 years.
